# Uterine inversion as an extremely rare cause of secondary infertility: A case report

**DOI:** 10.4274/tjod.65471

**Published:** 2015-03-15

**Authors:** Mehmet Sakıncı, Cem Yaşar Sanhal, Gayem İnayet Çelik, Şafak Olgan, Nasuh Utku Doğan

**Affiliations:** 1 Akdeniz University Faculty of Medicine, Department of Obstetrics and Gynecology, Antalya, Turkey

**Keywords:** infertility, uterus, inversion, myoma

## Abstract

Today, infertility takes a major place in gynecology practice. Non-puerperal uterine inversion is a rare event and usually accompanies submucous myoma, leiomyosarcoma, rhabdomyosarcoma, malignant mixed Mullerian tumour, and endometrial polyp. Herein, we report a 39-year-old woman who suffered from secondary infertility together with uterine inversion, which is an extremely rare co-existence.

## INTRODUCTION

Infertility is a condition of inability to achieve pregnancy in one year although a couple had regular and unprotected sexual intercourse. Today, infertility seems to be a common clinical problem. The worldwide prevalence among couples is about 13-15%^(1)^.

Uterine inversion is a rare event and it is generally seen just after vaginal birth^(2)^. Non-puerperal uterine inversion is even more rarely observed condition. Only 150 cases were reported between the years 1887 and 2006^(3)^. When the available literature is reviewed, it is seen that a substantial proportion of these patients had been diagnosed as having leiomyoma uteri in postmenopausal period or had malignancy. In this case report, we aimed to present a woman who suffered from secondary infertility together with uterine inversion which is an extremely rare co-existence.

## CASE

A 39-year-old, gravida 2 parity 1 patient applied to our clinic with the complaint of inability to achieve pregnancy, despite unprotected sexual intercourse for 4 years. She complained of prolonged and heavy menstrual bleeding sometimes accompanied by intermenstrual bleeding which was evident especially during the past 6 months. In past medical history, she had undergone diagnostic laparoscopy and hysteroscopy operation also due to infertility. In the operative note; it was reported that, during the laparoscopic examination; uterus was found to be bigger than normal, bilateral adnexal structures and tubal patency were unremarkable, during the hysteroscopic examination; there was a 4 cm submucous myoma in the fundal anterior wall of the uterus and this myoma has been removed hysteroscopically and the uterine cavity has been made sufficient for a probable pregnancy.

In bimanual and speculum examination, there was an approximately 10x7 cm hard mass lesion containing ulcerated-necrotic areas which filled the vagina entirely. Even effaced the cervix could not be observed or palpated. Uterine fundus could not be palpated during bimanual examination. Ultrasonographic examination revealed a 101x69x58 mm uterus with heterogenous echogenicty containing cystic structures up to 2 cm. Although endometrium was not clearly identified, bilateral adnexal structures were normal. Hemoglobin level of the patient was 11.7 g/dL and hormone panel was normal. With these findings and the preliminary diagnosis of uterine inversion, the patient underwent operation in lithotomy position.

**Figure 1:**
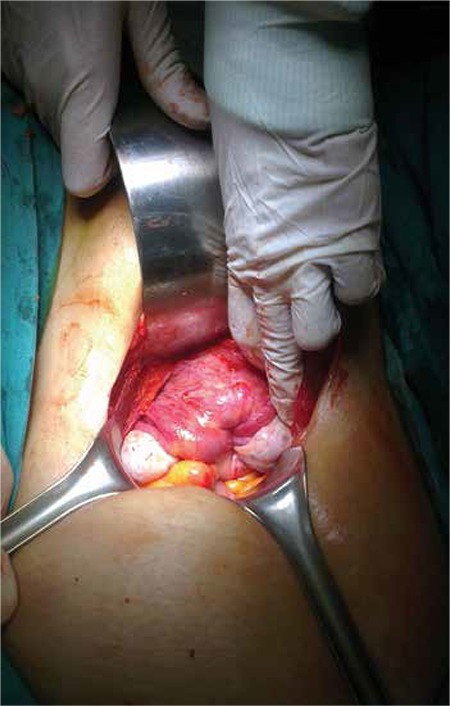
Appearance of completely inverted uterus at laparotomy

Under general anesthesia, we made attempts to reduce the inverted uterine mass but we were unsuccessful. Thereafter, we decided to perform laparotomy with Pfannenstiel incision. On exploration, inversion of the uterus was denoted. The fundus of the uterus was found to be collapsed through the direction of fallopian tubes (Figure 1). Other pelvic organs were normal. A submucous myoma on the left cornual region (4x2 cm) and two other submucous myomas at the fundus (3x2 cm, 2x2 cm) were excised transvaginally. The borders of the myomas were not clearly defined. Correction of the inverted uterus still could not be achieved even after myomectomies. Only the lower uterine segment could be partially reduced. The vesicouterine plica was dissected and the bladder base was removed. An anterior 3 cm uterine incision was accomplished. The depressed uterine fundus was reduced by means of index digit of the surgeon and holding with tenacula. The endometrium was very thin. Afterwards, the uterine incision was closed. The control of hemorrhage was done and a pelvic drain was placed. Two units of erythrocytes were given intraoperatively. The patient was discharged on the fifth postoperative day without any complication. Macroscopic pathology examination revealed a fragmented 10.5x6x3 cm contaminated white material containing partial hemorrhagic and swirling areas, and microscopic examination revealed adenomyoma and endometritis. The patient is on the sixth postoperative month without any complaints.

## DISCUSSION

The incidence of uterine inversion, which is one of the extremely rare entities in gynecology, is still not known. Although the certain etiology is not defined, some mechanisms, such as thin uterine wall, co-existent and rapidly growing tumor, fundal tumoral location, tumor with a thin pedicle, and cervical dilatation due to uterine cavity distention were put forward^(4)^. Until today, the most common tumor in benign group was myoma. On the other hand, the most common tumors in malignant group were sarcoma, endometrial carcinoma and malignant mixed Mullerian tumor^(5)^. In our case, the myomectomy procedure in the history of the patient together with the presence of the myomas excised during the operation point to the recurrent and rapidly growing myomas as the etiological factor.

The primary diagnosis mostly depends on patients’ symptoms and clinical findings. Increase in vaginal discharge, irregular bleeding and pelvic pain are the most common symptoms^(6)^. Nevertheless, infertility was the main problem in our case. Although non-palpable uterine fundus and inability to visualize the cervix on vaginal examination were clues for the diagnosis, most cases need to be evaluated by the additional methods including gynecological examination under general anesthesia^(7)^. Dealing with our case, the cervix was unidentified and the mass that fulfilled the vagina was visualized.

It was shown in the previous studies that magnetic resonance imaging is the most sensitive diagnostic tool for uterine inversion. Ultrasonography might reveal some clues (presence of myoma uteri, bilateral retracted ovaries to the midline, and uterine arteries not lateral but inside the uterine cavity detected by Doppler ultrasonography) as well^(7,8)^. However, magnetic resonance imaging was not used in our case as the examination and the ultrasonographic findings were satisfactory enough.

The management of these patients should be based on the underlying etiology. An acute puerperal inversion which might threaten the patients’ life needs either manual reduction or operative procedures described by Huntington or Haultain. Myomectomy or even hysterectomy (when inversion recurs in spite of myomectomy) might also be indicated in non-puerperal inversions. Subsequently, we performed vaginal myomectomy which provided both reduction of the uterus and preservation of the patient’s fertility.

The past medical history of the patient revealed hysteroscopic myomectomy which was performed two years ago. It was understood from the records that the resection of the myoma uteri (sub-mucous, diameter of 4 cm) resulted in a sufficient uterine cavity. Thus, we believe that a recent myoma uteri lead to the uterine inversion in our case. It should be kept in mind that incomplete endoscopic procedures might cause this pathology as well.

In this case report, we presented an atypical case of uterine inversion due to myoma uteri that resulted in infertility. Our case might provide evidence to fertility preserving options of uterine inversion by appropriate preoperative evaluation of patients’ symptoms and findings in an unusual population such as the patients with infertility.
